# Mosaicism for r(X) and der(X)del(X)(p11.23)dup(X)(p11.21p11.22) provides insight into the possible mechanism of rearrangement

**DOI:** 10.1186/1755-8166-1-16

**Published:** 2008-07-25

**Authors:** Oleg A Shchelochkov, M Lance Cooper, Zhishuo Ou, Sandra Peacock, Svetlana A Yatsenko, Chester W Brown, Ping Fang, Pawel Stankiewicz, Sau Wai Cheung

**Affiliations:** 1 Department of Molecular and Human Genetics, Baylor College of Medicine, Houston, Texas, USA

## Abstract

We report a patient with a unique and complex cytogenetic abnormality involving mosaicism for a small ring X and deleted Xp derivative chromosome with tandem duplication at the break point. The patient presented with failure to thrive, muscular hypotonia, and minor facial anatomic anomalies, all concerning for Turner syndrome. Brain MRI revealed mild thinning of the corpus callosum, an apparent decrease in ventricular white matter volume, and an asymmetric myelination pattern. Array comparative genome hybridization analysis revealed mosaicism for the X chromosome, deletion of the short arm of an X chromosome, and a duplication of chromosome region Xp11.21-p11.22. G-banded chromosome and FISH analyses revealed three abnormal cell lines: 46,X,der(X)del(X)(p11.23)dup(X)(p11.21p11.22)/46,X,r(X)(q11.1q13.1)/45,X. The small ring X chromosome was estimated to be 5.2 Mb in size and encompassed the centromere and Xq pericentromeric region. X chromosome inactivation (XCI) studies demonstrated a skewed pattern suggesting that the ring X remained active, likely contributing to the observed clinical features of brain dysmyelination. We hypothesize that a prezygotic asymmetric crossing over within a loop formed during meiosis in an X chromosome with a paracentric inversion resulted in an intermediate dicentric chromosome. An uneven breakage of the dicentric chromosome in the early postzygotic period might have resulted in the formation of one cell line with the X chromosome carrying a terminal deletion and pericentromeric duplication of the short arm and the second cell line with the X chromosome carrying a complete deletion of Xp. The cell line carrying the deletion of Xp could have then stabilized through self-circularization and formation of the ring X chromosome.

## Background

Turner syndrome is diagnosed in 1 of 5,000 live female births. Six to fifteen percent of patients with Turner syndrome are mosaic carriers of a ring X chromosome. [1,2]. Several studies in the last decade have made significant contributions to our understanding of the clinical phenotype in patients with r(X) [2-7]. It has been demonstrated that most individuals carrying an r(X) chromosome show somatic signs of Turner syndrome, including short stature, peripheral edema, characteristic facial features, low posterior hairline, ovarian dysgenesis, endocrine disorders, and autoimmune conditions. At the same time, patients with r(X) can be at a higher risk for other findings, including mental retardation, learning difficulties, autistic spectrum disorders, and structural brain abnormalities [4,8,9]. These additional features can be partially explained by the status of X inactivation, size, origin, and replication timing of the ring, genes affected by copy number variations, and the percent of mosaicism [3,6,8,10]. Dennis et al. (2000) concluded that severe r(X) phenotype can be seen in patients with active, large r(X) chromosomes lacking the *XIST *locus. Possible submicroscopic duplications in active ring X chromosomes can go undetected by chromosomal analysis and may encompass dosage sensitive genes, leading to functional disomy or even trisomy, and resulting in clinically relevant phenotypes as has been demonstrated in autosomes [11]. Additionally, a ring chromosome comprised of Y-chromosomal material should be distinguished from that containing X-derived material. Early detection of Y-derived ring chromosomes is necessary since individuals with r(Y) and Turner syndrome are at risk to develop gonadoblastoma, immature teratoma, and endodermal sinus carcinoma, conditions preventable by prophylactic gonadectomy [12]. Special attention has been given also to the possible association between r(X) and Kabuki syndrome [5,13,14]. McGinniss et al (1997) have hypothesized that overexpression of dosage sensitive genes from an X chromosome lacking *XIST *is responsible for some cases with Kabuki syndrome-like features.

Several methods have been used to characterize ring X chromosomes including fluorescence *in situ *hybridization (FISH), cell replication studies, real time PCR of *XIST *expression, and the *AR *locus methylation status. Array comparative genomic hybridization (aCGH) is a novel, clinically validated method that can be applied to studying r(X) chromosomes. CGH microarrays have been used for detection of clinically relevant chromosomal abnormalities in a variety of postnatal patient samples [15-19]. Array CGH adds significantly to our ability to characterize the origin and size of a ring chromosome, to detect chromosomal mosaicism, and to identify additional microscopic deletions or duplications [20-23]. By providing important information about the structural features of ring chromosomes, array CGH also gives new insight into our understanding of the mechanisms of ring formation.

We present a patient with clinical features of Turner syndrome and a complex X chromosome rearrangement, which was detected by chromosomal microarray analysis, and further characterized by G-banding chromosome analysis and X chromosome inactivation studies.

## Clinical Presentation

A 10-month-old girl (RX1) was referred to the genetics consultation service for evaluation of failure to thrive, muscular hypotonia, and delayed closure of the anterior and posterior fontanels. She was born at 32 weeks gestation with normal growth parameters, when adjusted for gestational age. Evaluation at 10 months of age revealed delayed growth parameters. Her weight and length were 3 standard deviations (SD) below the mean, and head circumference was at the 95^th ^centile. At age 13 months, she had no signs of gross motor, fine motor and or speech delays. Her physical exam was remarkable for mild dysmorphic features, including downslanting palpebral fissures, wide nasal bridge, broad forehead, and down-turned corners of the mouth. The previous workup for failure to thrive included a non-diagnostic comprehensive metabolic panel, skeletal survey and CT brain imaging. Echocardiogram and renal ultrasound were normal. At age 2 2/12 years she developed seizures. Brain imaging revealed mild thinning of the corpus callosum, a decrease in ventricular white matter volume, punctate enhancement in the right caudate head likely representing a vascular malformation, and an asymmetric myelination pattern within the left superior temporal lobe, raising the possibility of cortical dysplasia. Her other current medical problems include short stature, recurrent otitis media and adenoid hypertrophy. Clinical findings and their comparison to other reports of r(X) are summarized in Table 1.

**Table 1:** Comparison of the clinical findings between patient RX1 and previously reported patients with r(X).

**Findings**	**Case**	**45,X/46,X,r(X)**^2^	**Reference**
	
	*RX1*		
*Constitutional symptoms*			
Short stature/failure to thrive	+	98%	[6]
Special education/Developmental delay	-	33–52%	[3,6]
Kabuki syndrome	-	13%	[3,4]

*Craniofacial features*			
High arched palate	-	34%	[3]
Microcephaly	-	29%	[3]
Micrognathia	-	R^1^	[8,30]
Thin upper lip	-	R	[31]
*ENT*			
Recurrent and chronic otitis media	+	62%	[3]
*Eyes*			
Strabismus	-	R	[6,8]

*Neck*			
Neck webbing	-	4%	[3]
Low posterior hairline	-	19%	[3]

*Cardiovascular*			
Bicuspid aortic valve	-	15%	[31]
Aortic valve disease	-	5%	[31]
Aortic coarctation	-	4%	[31]
*Endocrine system*			
Hypothyroidism	-	10%^3^	[32]

*Skeletal system*			
Short metacarpal bones	-	23%	[3]
Postaxial polydactyly	-	R	[8]

*Genitourinary system*			
Ovarian dysgenesis/ovarian failure	n/a	40%	[3]

*Skin*			
Hypomelanosis of Ito	-	2–6%	[3,6]
Syndactyly	-	15%	[6]
Lymphedema	-	18%	[6]
Hypertrichosis/hirsutism	-	R	[13,33,34]

*Autoimmune disorders*	-	9%	[3]

*Neurologic findings*			
Seizures	+	10%	[6]

^1^"NR" indicates the finding was not reported and "R" indicates the finding was reported, but the percentage of patients is not known; "n/a" indicates that the data is not available.

^2^The percentages were quoted or calculated from the provided references.

^3^Percent of patients with autoimmune thyroid disease.

## Results

**Figure 1:**
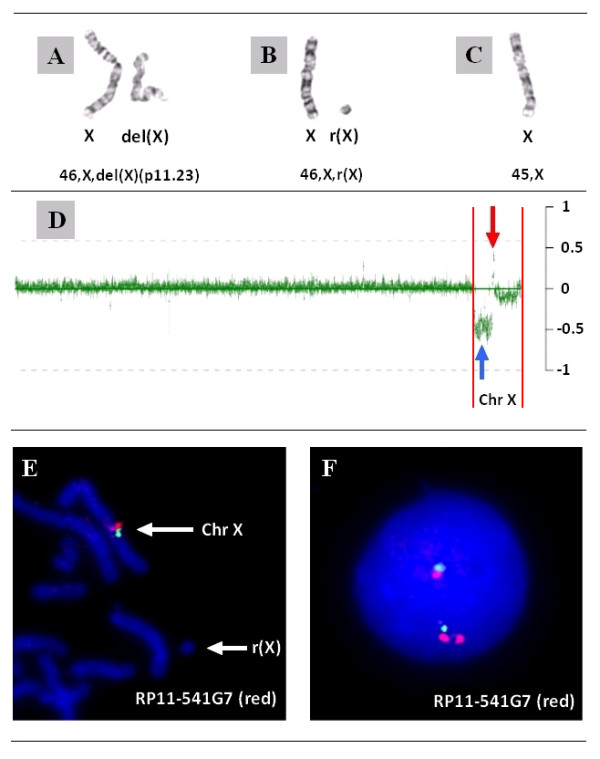
**Chromosome and CMA findings in patient RX1**. **A-C**. Partial karyotype demonstrating three cell lines: 46,X,del(X)(p11.23)/46,X,r(X)/45,X. **D**: A whole genome plot of the chromosomal microarray analysis showing a loss in copy number of clones between RP11-1325A17 and RP11-416B14 of the short arm of chromosome X (blue arrow) and a gain in copy number of clones (RP11-258C19, RP11-541G7 and RP11-465B24) corresponding to the Xp pericentromeric region (red arrow). The start and end of the X chromosome is highlighted by two red lines. **E**: Two probes RP11-541G7 (red signal) and RP11-52N6 (green signal) corresponding to the duplicated region on array CGH can be seen on the normal X chromosome with the r(X) chromosome lacking hybridization. The hybridization to the der(X) is not shown. **F**: A duplication of the Xp11.21-p11.22 region detected on the interphase cell using RP11-541G7 probe inferred to originate from the der(X).

Array CGH using BAC/PAC suggested mosaicism for the X chromosome, which was confirmed by G-banding chromosome analysis showing the presence of three abnormal cell lines: 46,X,del(X)(p11.23)/46,X,r(X)/45,X (Figure 1A–C). In the first cell line, 46,X,del(X)(p11.23), a deletion of the short arm of the X chromosome at band Xp11.23 encompassed approximately 48 Mb (Figure 1D). The breakpoint mapped between probes RP11-1325A17 and RP11-416B14 at band Xp11.23. In the second cell line, 46,X,r(X), the ring X chromosome was estimated to be 5.2 Mb in size and encompassed the pericentromeric region on the long arm of chromosome X from cen(X) to Xq13.1. In the third cell line, monosomy X was found in a single cell. Monosomy X was confirmed by FISH in additional cells. In addition, Chromosome Microarray Analysis (CMA) detected a significant gain in copy number of material from Xp11.21-p11.22 detected by several clones, including RP11-258C19, RP11-541G7 and RP11-465B24 (Figure 1D). FISH analysis using the RP11-541G7 probe showed that the duplicated region was present in the pericentromeric region of the short arm of the chromosome del(X)(p11.23) and not in the ring X chromosome (Figure 1E and 1F). The karyotype was revised as follows: 46,X,der(X)del(X)(p11.23)dup(X)(p11.21p11.22)[9]/46,X,r(X)(q11.1q13.1)[8]/45,X[1].

**Figure 2:**
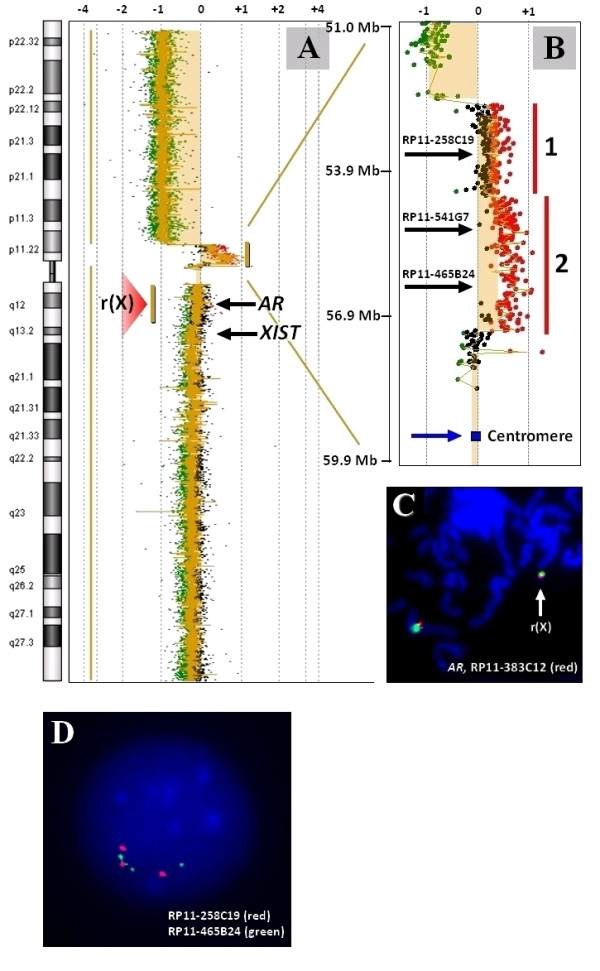
**Results of the 244 K oligonucleotide Agilent array in case RX1**. **A**: A graph representation of the 244 K Agilent oligonucleotide array for case RX1 demonstrates a gain in copy number of probes covering the pericentromeric region of chromosome band Xp11. **B**: The chromosome band Xp11 is enlarged and shows two segments of log_2 _values: area 1 and area 2. **C**: Red signal from RP11-383C12 corresponding to *AR *locus can be found on both the structurally normal X chromosome and ring X in the 46,X,r(X) cell line. **D**: An interphase demonstrating tandem duplication of RP11-258C19 and RP11-465B24 on the derivative X chromosome.

High resolution 244 K oligonucleotide microarray confirmed the gain in copy number of a segment corresponding to pericentric region Xp11.22-p11.21 (Figure 2A). The duplication was determined to be 4.6 Mb in size.

**Figure 3:**
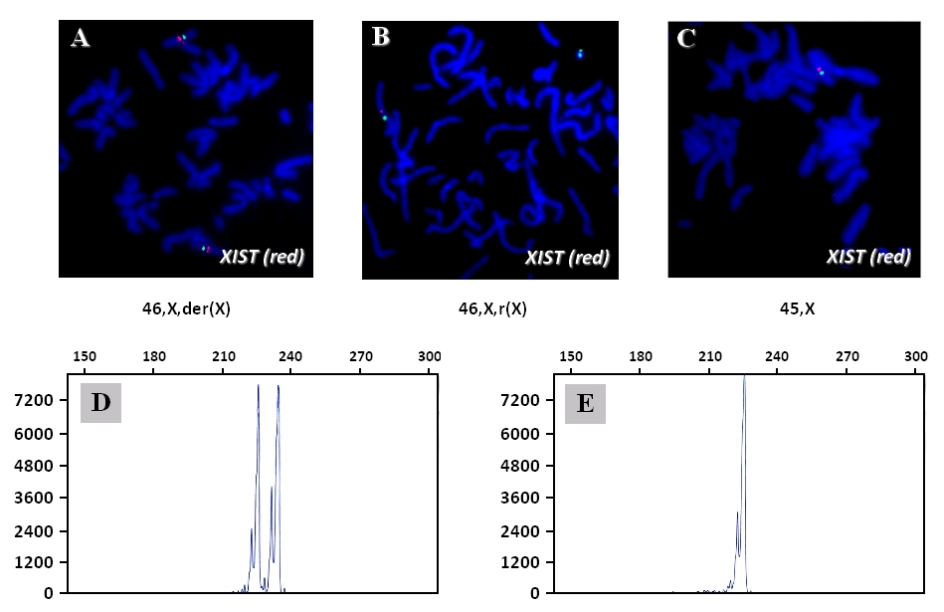
***XIST *FISH and results of the X chromosome inactivation assay**. **A-C**: FISH analysis using a *XIST *probe (red signal) demonstrates the presence of two *XIST *loci in 46,X,der(X) and one *XIST *locus in cell lines 46,X,r(X) and 45,X. The green signal represents a centromere probe (Cen X). **D**: X chromosome inactivation study shows two *AR *alleles in the peripheral lymphocytes without *Hpa*II digestion. **E**: XCI analysis shows only one *AR *allele after digestion with a methylation-sensitive restriction enzyme *Hpa*II.

The duplicated region had two segments with a bimodal distribution of log_2 _ratios (Figure 2B) referred to as regions 1 and 2. The log_2 _ratio corresponding to chromosome Xq11.1-q13.1 approximated closer to zero, suggesting a location on the ring X chromosome. This finding was confirmed by FISH, which demonstrated the presence of an *AR*-specific signal on r(X) (Figure 2C). Twenty of 100 analyzed ring X chromosomes were found to be dicentric (Figure 3B, green signal).

BAC clones RP11-258C19 (red) and RP11-465B24 (green), corresponding to regions 1 and 2 (Figure 2B), respectively, were used to demonstrate the "red-green-red-green-green" signal pattern in interphase cells (Figure 2D), indicating the direct orientation of the duplication. The metaphase images did not provide sufficient resolution to define the orientation of the tandem duplication relative to the telomere (data not shown). The presence of three green signals (probe RP11-465B24) and two red signals (probe RP11-258C19) on one of the X chromosomes was in agreement with the 244 K array data, consistent with the bimodal distribution of log_2 _ratios in regions 1 and 2 (Figure 2B).

FISH analysis with a *XIST*-specific probe showed the presence of two signals in cell line 46,X,der(X) (Figure 3A), one *XIST *signal in cell lines 46,X,r(X) (Figure 3B) and 45,X, indicating that *XIST *is not present on the r(X) (Figure 3C). X inactivation (XCI) analysis revealed a 100% skewing of the *AR *gene: two *AR *alleles prior to digestion with *Hpa*II (Figure 3D) and only one *AR *allele after digestion with methylation-sensitive restriction enzyme *Hpa*II (Figure 3E). This suggested that the only cell line that has an inactive X chromosome is the cell line with the der(X) and it was preferentially inactivated and that the r(X) chromosome was presumably active.

G-banding chromosome analysis of the mother revealed no abnormalities. An additional maternal blood sample to perform FISH using probes specific to Xp11.2 was not available. A paternal sample was not available for analysis.

## Discussion

This report presents the array CGH and cytogenetic findings in a patient with Turner syndrome due to mosaic r(X) and derivative chromosome with a deleted Xp segment and a tandem duplication of bands Xp11.21-p11.22 at the breakpoint. This combination has not been previously reported. The patient's phenotype at the time of her most recent evaluation at age 26 months included small stature, delayed bone maturation, diffuse muscular hypotonia, seizures, and mild developmental delay. Brain MRI revealed mild thinning of the corpus callosum, an apparent decrease in ventricular white matter volume, and an asymmetric myelination pattern. Of note, there were no features of Kabuki syndrome, a condition previously reported in some patients with r(X) [5,13,14]. The most plausible explanation for the brain imaging abnormalities is the overexpression of dosage sensitive genes from the ring X chromosome, which lacks *XIST *and thus presumably remains active. One of the genes present on the ring chromosome, *OPHN1*, is known to participate in neuron morphogenesis. When overexpressed *in vitro*, it can interfere with the actin cytoskeleton, playing a crucial role in axon growth and guidance, dendrite elaboration, and synapse formation [24].

Array CGH studies detected and provided detailed characterization of the copy number abnormality on chromosome X. The duplication of bands Xp11.21p-11.22, that escaped detection by routine chromosome analysis, was readily seen on the BAC-clone targeted chromosomal microarray and the high resolution oligoarray analyses (Figure 1D, Figure 2A and 2B). The array data was confirmed by FISH, which also localized the duplicated region to the derivative chromosome, and not the r(X) (Figure 3B).

The finding of a duplicated region associated with terminal deletions in a ring chromosome has been described previously [20,21]. Many of the reported cases had an inverted duplication at the breakpoint. Rossi et al (2007) identified two patients with concurrent deletion and inverted contiguous duplication (inv dup del) in the ring chromosomes and proposed that such abnormality could be the result of recombination between homologous segmental duplications located at the rearrangement's breakpoints leading to an intermediate dicentric chromosome followed by asymmetric breakage of the dicentric. According to this model, inv dup del undergoes an additional step of self-circularization for stabilization of r(X).

Our case is different in two ways: (1) the duplication is tandem and (2) the duplication is present in the non-circular chromosome in the presence of a ring chromosome. The formation of tandem duplication at the terminal breakpoint cannot be explained by concurrent deletion and reunion at Xp, since it would have resulted in inverted duplication, suggesting a different mechanism. Wolff et al (1996) concluded that chromosome region Xp11.2 is prone to breakage and reunion events between sister chromatids or homologous X chromosomes, and could be responsible for the majority of i(Xq) monocentrics or dicentrics. The pericentromeric region of Xp is particularly rich in low copy repeats, thought to be responsible for six classes of i(Xq) originating from breakpoints spanning Xp11.21p11.22 [25].

**Figure 4:**
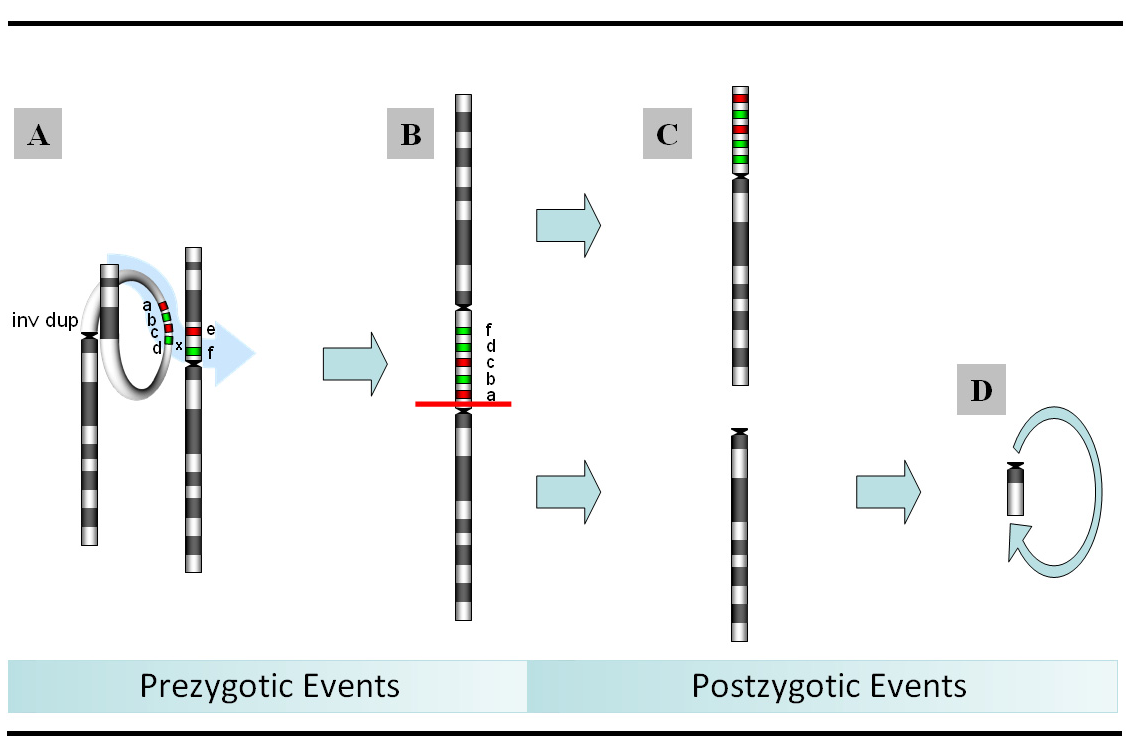
**The proposed mechanism of r(X) and i(Xq) formation**. **A**: During the pachytene phase of meiosis I, a clinically silent inverted and duplicated segment X p11.1-p11.22 (green arrow) can form a loop with a normal X chromosome. Asymmetric crossing over within the loop may lead to the formation of dicentric chromosome with tandem duplication between the centromeres. **B**: After the zygote is formed, the dicentric chromosome could undergo fission with the formation of two cell lines. **C**: The cell retaining chromosome with terminal deletion and pericentromeric duplication of the short arm results in cell line 46,X,der(X)del(X)(p11.23)dup(X)(p11.21p11.22). **D**: The other cell retaining chromosome X with a complete deletion of the short arm results in cell line 46,X,r(X)(q11.1q13.1). **E**: Mitotically unstable del(Xp) could undergo stabilization through postzygotic terminal deletion of the long arm with self-circularization resulting in cell line r(X).

The three cells lines in our patient may have resulted from the instability of an intermediate dicentric chromosome formed prezygotically. The presence of a complex tandem duplication-triplication in Xp11.21p11.22 (Figure 2D) led us to propose a model of der(X) and r(X) formation, in which one of the parents may be a heterozygous carrier for a clinically silent duplicated paracentric inversion of chromosome band Xp11.1p11.22 (Figure 4). During the pachytene phase of meiosis I, the inverted segment can form a loop with a normal X chromosome (Figure 4A). Asymmetric crossing over mediated by low copy repeats within the loop may lead to the formation of a dicentric chromosome with tandem duplication-triplication between the centromeres (Figure 4B). In the early postzygotic period, the dicentric chromosome could have undergone a breakage, wherein some of the cells retained a chromosome with terminal deletion and pericentromeric duplication of the short arm (Figure 4D), while other cells retained a chromosome X with a complete deletion of the short arm (Figure 4D). The cell line with del(Xp) could have undergone stabilization through postzygotic terminal deletion of the long arm with self-circularization and formation of r(X) (Figure 4E). Finally, due to mitotic instability, some of the abnormal X chromosomes were lost resulting in third cell line 45,X. Unfortunately, parental chromosomes were not available for further analysis to confirm this hypothesis.

## Conclusion

The reported unique and complex cytogenetic abnormality involving mosaicism for r(X) and deleted Xp derivative chromosome with tandem duplication at the break point provides insight into the possible mechanism of der(X) and r(X) formation. We suggest that *XIST*-mediated silencing, size of r(X) and i(Xq), gene content and the level of mosaicism all play important roles in determining phenotypic outcomes. The parents of patients with r(X) and derivative chromosomes with terminal duplications should be offered evaluation using array CGH.

## Materials and methods

### Human Subjects

Following the initial result of the chromosomal microarray analysis, an informed consent for the whole genome array-based oligonucleotide CGH study was obtained from the mother. The research protocol was approved by the Institutional Review Board (IRB) for Baylor College of Medicine (BCM) and Affiliated Hospitals.

### FISH Analysis

Chromosome and FISH analyses were performed on peripheral blood lymphocytes using standard procedures. Briefly, the BAC clone of interest was grown in TB media with 20 μg/ml chloramphenicol. DNA was extracted from bacterial artificial chromosome (BAC) clones (Eppendorf Plasmid Mini Prep kit, Hamburg, Germany) and directly labeled with SpectrumOrange™ dUTP by nick translation (Vysis, Downer Grove, IL) according to the manufacturers' instructions. Digital FISH images were captured by a Power Macintosh G3 System and MacProbe version 4.4 (Applied Imaging; San Jose, CA).

### Array-CGH analysis

The patient's DNA was extracted from whole blood using the Puregene DNA extraction kit (Gentra, Minneapolis, MN) according to the manufacturer's instructions and studied by aCGH using the BAC/PAC microarray platform (CMA V6.1) [26,27] and the 244 K whole genome oligonucleotide microarray (Agilent, Santa Clara, CA). The BAC/PAC microarray includes 1472 BAC and PAC clones, covers ~150 genomic disorders with minimum backbone coverage of every chromosome at the 650-band level of cytogenetic resolution . The procedures for probe labeling and hybridization of our BAC arrays were reported previously [26,27]. For the oligonucleotide microarray study, DNA was digested, labeled, and hybridized according to the manufacturer's instructions, with some modifications [28]. The slides were scanned into image files using a GenePix Model 4000B microarray scanner (Molecular Devices, Sunnyvale, CA) or an Agilent G2565 laser scanner. Microarray image files of oligonucleotide microarrays were quantified using Agilent Feature extraction software (v9.0), and text file outputs from the quantification analysis were imported either into the Agilent CGH Analytics software program or converted to BAC-level emulation data by combining oligo data corresponding to regions encompassed by BAC clones ("emulated BAC clone") and then using custom-written analysis software package for copy number analysis, as described previously [16,27].

### X chromosome inactivation (XCI)

X-inactivation studies were performed according to a previously described protocol with some modifications [29]. Briefly, two sets of reactions were set-up for each female, including 100 ng of genomic DNA digested with the methylation-sensitive restriction enzyme *Hpa*II (NewEngland Biolabs, Ipswich, MA); and another 100 ng DNA incubated with 1× enzyme buffer only. PCR primers flanking the androgen receptor (*AR*) CAG repeat region were as follows: 5'-ACCAGGTAGCCTGTGGGGCCTCTACGATGGGC-3' (forward) and 5'-CCAGAGCGTGCGCGAAGTGATCCAGAACCCGG-3' (reverse). PCR amplification was performed using 0.5 ng from each DNA sample. PCR products were analyzed using capillary electrophoresis on the 3100 ABI Automated DNA Sequencer with GeneMapper software. Random X chromosome inactivation is expected to produce two signals if each chromosome has a different *AR *allele and an intact *XIST *locus. Non-random inactivation of an X chromosome or the absence of an *AR *allele in a deleted chromosome will result in PCR amplification of only one allele after digestion with *Hpa*II.

## Consent

Written informed consent was obtained from the parent for publication of this case report. A copy of the written consent is available for review by the Editor-in-Chief of this journal.

## Competing interests

The authors declare that they have no competing interests.

## Authors' contributions

OAS conceived the study, participated in the initial diagnosis, follow-up care, performed parts of the cytogenetic analysis, and drafted the manuscript. MLC performed cytogenetic analysis and participated in the study design. ZO conducted molecular characterization using array CGH. SP participated in the recruitment of the patient and correction of the manuscript. SAY analyzed the array CGH data and participated in the design of the study. CWB participated in the initial diagnosis and follow-up cares and helped to draft the manuscript. PF provided data on the X chromosome inactivation. PS participated in the study design and coordination and helped to draft the manuscript. SWC conceived of the study, and participated in its design and coordination and helped to draft the manuscript. All authors read and approved of the final manuscript.
